# Supplementary material for: Genetic Diversity, Evolutionary Dynamics, and Ongoing Spread of Pedilanthus Leaf Curl Virus
Source: Viruses. 2023 Nov 30;15(12):2358. doi: 10.3390/v15122358 (PMC10747432; doi:10.3390/v15122358)
Supplement: Supplementary file 1 [file viruses-15-02358-s001.zip › viruses-2714703-supplementary.pdf]

Supplementary data

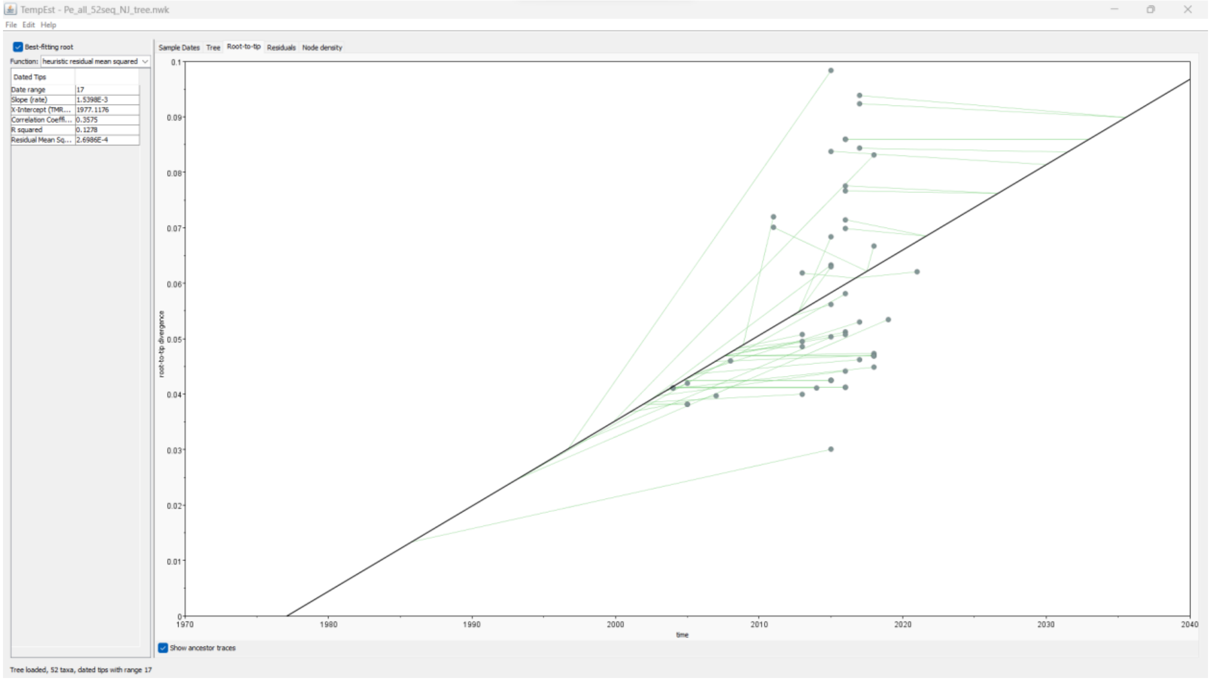


**Figure S1.** TempEST analysis of PeLCV-all to assess potential evolutionary time of the most recent common ancestor (MRCA)

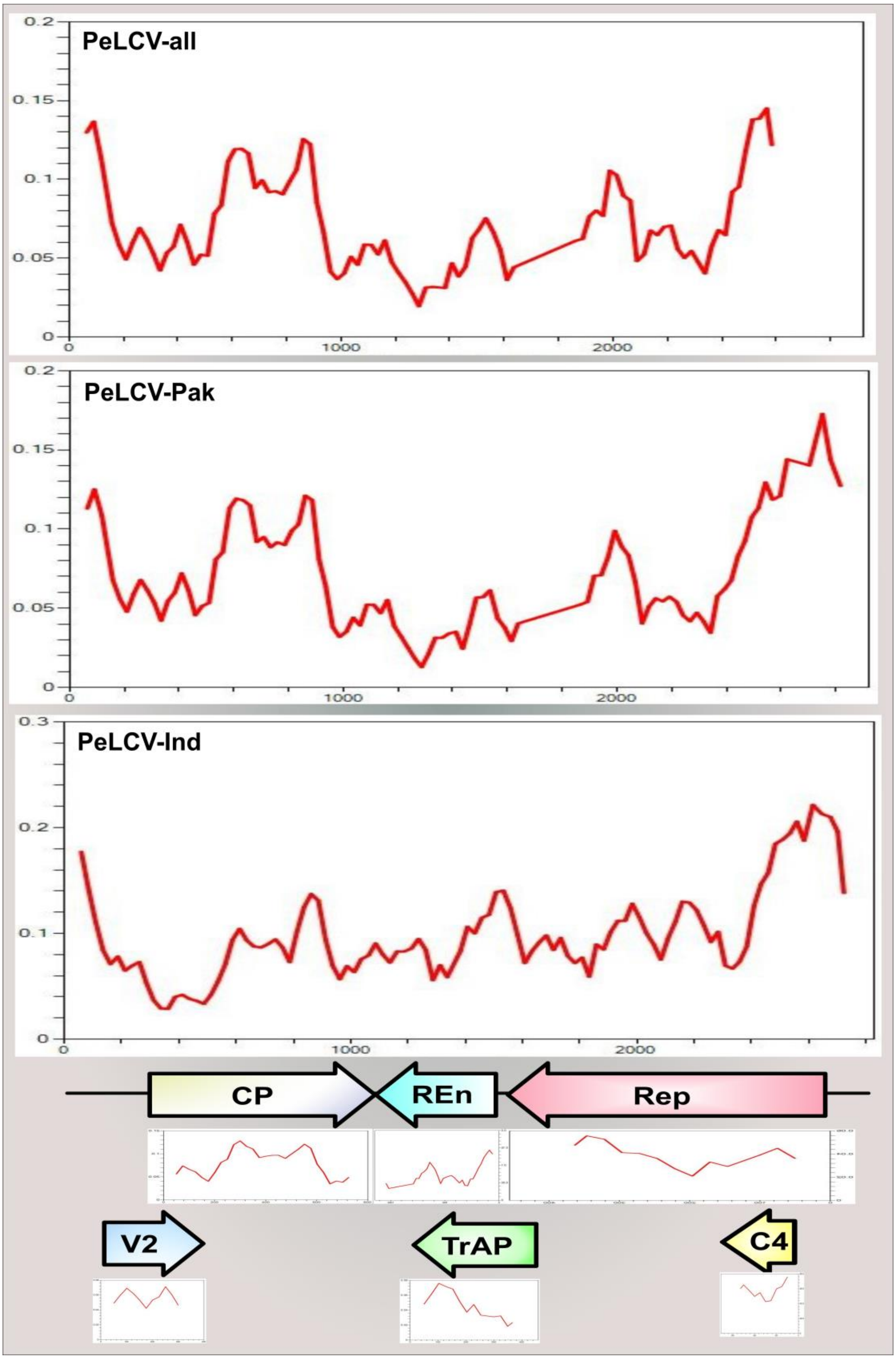


**Figure S2:** Total nucleotide diversity across all the nucleotide of all the PeLCV datasets. Along y-axis nucleotide diveristy (π) and along x-axis nucleotide position is mentioned. A linear genome organization is mentioned to map the π value at each ORF.

**Table S1.** Recombination events detected in the entire PeLCV-all population

| **PeLCV accession #** | **Number of recombination events** | **Recombination breakpoint*** | **Major parent** | **Minor parent** | **RDP methods** | **Highest P values detected by the underline method#** |
|---|---|---|---|---|---|---|
| AM712436 | 2 | 2738-16 | JN807764 | NC008299 | R,G,B,M,**C**, 3S | 80.4E−07 |
| | | 2576-2625 | NV008299 | MN566097 | R,**G**,M,C,3S | 6.42E−04 |
| AM948961 | 1 | 1967-2402 | HF568781 | KY937947 | R,**M**,C,3S | 1.67E−04 |
| DQ116884 | 2 | 1138-1661 | Unknown | MN885483 | R,B,M,C,S,**3S** | 1.82E−05 |
| | | 1746-2559 | Unknown | AM848961 | R,G,B,**M**,C,S,3S | 6.50E−11 |
| JN807764 | 3 | 730-1924 | JQ012790 | MN910265 | B,**M**,C,3S | 1.71E−17 |
| | | 1956-2476 | KY937947 | MN885482 | R,M,C,**3S** | 2.93E−08 |
| | | 2600-2625 | NC008299 | MN566097 | R,**G**,M,C,3S | 6.42E−04 |
| JQ12790 | 2 | 1166-2404 | KY937947 | MH550115 | R,G,B,M,S, **3S** | 8.66E−13 |
| | | 2600-2625 | NC008299 | MN566097 | R,**G**,M,C,3S | 6.42E−04 |
| KX168427 | 2 | 2746-364 | JN807764 | NC008299 | R,G,B,M,**C**,3S | 8.04E−07 |
| | | 2576-2625 | NC008299 | MN566097 | R,**G**,M,C,3S | 6.42E−04 |
| KX671561 | 1 | 1972-2747 | MG764705 | MN566097 | G,B,M,**S**,3S | 2.37E−08 |
| KX671562 | 1 | 1740-2658 | MG764705 | MN566097 | G,B,M,**S**,3S | 2.37E−08 |
| KX671563 | 2 | 1679-2437 | LN713272 | Unknown | R,G,M,C,S,**3S** | 4.56E−18 |
| | | 1865-1963 | KX671562 | Unknown | G,M,**S**,3S | 6.01E−13 |
| KX710160 | 2 | 834-1075 | MN885483 | Unknown | **R**,G,B,M,C,S,3S | 1.55E−09 |
| | | 2559-648 | LN713272 | KX168427 | R,G,**M**,C,3S | 1.26E−09 |
| KX711622 | 1 | 1731-2664 | MG764705 | MN566097 | G,B,M,**S**,3S | 2.37E−09 |
| KY937947 | 2 | 2576-2625 | NC008299 | MN566097 | R,**G**,M,C,3S | 6.42E−04 |
| | | 2738-438 | JN807764 | NC008299 | R,G,B,M,**C**,3S | 8.04E−07 |
| KY978406 | 2 | 2576-2625 | NC008299 | MN566097 | R,**G**,M,C,3S | 6.42E−04 |
| | | 2738-438 | JN807764 | NC008299 | R,G,B,M,**C**,3S | 8.04E−07 |
| LN678638; LN713272; LN713273 | 1 | 1965-2427 | HF568781 | KY937947 | R,M,**C**,3S | 1.07E−04 |
| LT600729 | 3 | 45-1041 | HF568781 | Unknown | R,G,B,M,C,**3S** | 1.58E−21 |
| | | 1964-2385 | MG764705 | Unknown | R,M,C,**S**,3S | 7.56E−15 |
| | | 2608-1272 | HF568781 | Unknown | G,B,M,C,S,**3S** | 2.15E−60 |
| LT795117 | 2 | 1956-2385 | MG764705 | Unknown | R,M,C,**S**,3S | 7.56E−15 |
| | | 2505-1280 | HF568781 | Unknown | G,B,M,C,S,**3S** | 2.15E−60 |
| LT795118 | 3 | 45-1148 | HF568781 | Unknown | R,G,B,M,C,S,**3S** | 1.58E−21 |
| | | 1956-2385 | MG764705 | Unknown | R,M,C,**S**,3S | 7.56E−15 |
| | | 2505-1280 | HF568780 | Unknown | G,B,M,C,S,**3S** | 2.15E−60 |

| | | | | | | |
|---|---|---|---|---|---|---|
| MF135486 | 1 | 52-1956 | MF135486 | AM948961 | G,M,C,**3S** | 3.03E−09 |
| MF278788; MF278789 | 2 | 2576-2625 | NC008299 | MN566097 | R,**G**,M,C,S,3S | 6.42E−04 |
| | | 2738-438 | JN807764 | NV008299 | R,G,B,M,**C**,3S | 8.04E−07 |
| MG764701; MG764702; MG764703:MG764705 | 1 | 1869-2289 | HF568781 | KY937947 | R,**M**,C,3S | 1.67E−04 |
| MH550115 | 4 | 638-1145 | Unknown | LN678638 | R,G,M,C,**S** | 6.63E−18 |
| | | 1202-1598 | OM993557 | KX671562 | R,M,C,**S**,3S | 2.06E−12 |
| | | 1519-2017 | HF568781 | KY937947 | **R**,G,M,C,3S | 3.39E−05 |
| | | 2753-273 | MK158208 | KX671562 | **R**,G,B,M,C,S,3S | 9.64E−12 |
| MH643736 | 2 | 1201-2398 | KY937947 | MH550115 | R,G,B,M,C,**3S** | 8.66E−13 |
| | | 2600-2625 | NC008299 | MN566097 | R,**G**,M,C,3S | 6.42E−07 |
| MH765695 | 3 | 116-570 | MF278789 | Unknown | R,G,M,C,S,**3S** | 1.30E−08 |
| | | 2577-2625 | NC008299 | MN566097 | R,**G**,M,C,3S | 6.42E−04 |
| | | 2741-600 | JN807764 | NC008299 | R,G,B,M,**C**,3S | 8.04E−07 |
| MH973686 | 2 | 1956-2385 | MG764705 | Unknown | R,M,C,**S**,3S | 7.56E−15 |
| | | 2505-1280 | HF568781 | Unknown | G,B,M,C,S,**3S** | 2.15E−60 |
| MH973687 | 3 | 1745-1984 | KX710160 | Unknown | G,M,C,**S**,3S | 1.59E−09 |
| | | 1985-2707 | Unknown | MF135486 | R,M,C,S,**3S** | 5.73E−10 |
| | | 2724-1103 | HF568781 | Unknown | R,G,B,M,C,S,**3S** | 1.58E−21 |
| MH973688 | 3 | 1743-1985 | KX710160 | Unknown | G,M,C,**S**,3S | 1.59E−09 |
| | | 1994-2678 | Unknown | MF165486 | R,M,C,S,**3S** | 5.73E−11 |
| | | 2750-1168 | HF568781 | Unknown | R,G,B,M,C,S,**3S** | 1.58E−21 |
| MK158207; MK158208 | 3 | 639-1140 | Unknown | LN678638 | R,G,M,C,S,**3S** | 6.63E−18 |
| | | 1141-1659 | OM993557 | LN713272 | **R**,G,B,M,C,S,3S | 1.01E−09 |
| | | 1705-2416 | HF568781 | KY937947 | R,G,B,M,C,S,3S | 1.01E−09 |
| MK158209 | 3 | 384-2147 | OM993555 | Unknown | R,G,B,M,C,S,**3S** | 2.76E−16 |
| | | 1484-1974 | OM993555 | Unknown | R,G,M,C,S,**3S** | 2.54E−11 |
| | | 2393-2696 | MH550115 | MH973687 | G,B,M,C,S,**3S** | 6.48E−28 |
| MN566097; MN566098 | 2 | 381-511 | LN313272 | KY937947 | **R**,G,M,C,S,3S | 8.61E−12 |
| | | 1968-2389 | MN566097 | Unknown | R,M,C,**S**,3S | 7.56E−15 |
| MN885484 | 2 | 415-1678 | KX671562 | MH973687 | R,G,B,M,C,S,**3S** | 4.31E−43 |
| | | 415-1152 | LN713272 | OM993557 | M,C,S,**3S** | 1.02E−14 |
| MN885485 | 2 | 423-1694 | KX671562 | MH973687 | R,G,B,M,C,S,**3S** | 4.31E−43 |
| MN910265 | 4 | 272-812 | MF278789 | Unknown | R,G,B,M,C,S,**3S** | 7.93E−13 |
| | | 2120-2443 | KY937947 | Unknown | **R**,G,B,M,C,S,3S | 4.63E−11 |
| | | 2576-2625 | NC008299 | MN566097 | R,**G**,M,C,3S | 6.49E−04 |
| | | 2738-271 | JN807764 | NC008299 | R,G,B,M,**C**,3S | 8.05E−07 |
| NC008299 | 2 | 1138-1661 | Unknown | MN885483 | R,B,M,C,**S** | 9.12E−08 |
| | | 1662-2564 | Unknown | AM948961 | R,G,B,**M**,C,S | 6.95E−11 |

| | | | | | | |
|---|---|---|---|---|---|---|
| NC012118 | 1 | 2762-438 | JN807764 | NC008299 | R,G,B,M,**C**,3S | 8.04E−07 |
| OM144969 | 1 | 1277-2467 | HF568781 | Unknown | G,B,M,C,**3S** | 1.34E−45 |
| OM993555 | 3 | 926-1472 | OM993557 | MK158209 | R,G,B,M,C,S,**3S** | 1.04E−09 |
| | | 1512-2009 | HF568781 | KY937947 | **R**,G,M,C,S,3S | 3.39E−06 |
| | | 2328-254 | MK158208 | MF278789 | B,M,C,S,**3S** | 2.38E−12 |
| OM993556 | 5 | 636-1143 | Unknown | LN678638 | R,G,M,C,**S** | 6.63E−18 |
| | | 1376-1598 | OM993557 | KX671562 | R,M,C,**S**,3S | 2.06E−12 |
| | | 1535-2405 | HF568781 | KY937947 | **R**,G,M,C,S,3S | 3.39E−06 |
| | | 2512-2784 | Unknown | MK158208 | R,G,B,M,C,S,**3S** | 1.86E−25 |
| | | 2785-304 | MK158208 | KX671562 | **R**,G,B,M,C,S,3S | 9.63E−12 |
| OM993557 | 3 | 636-1144 | Unknown | LN678638 | R,G,M,C,**S** | 6.63E−18 |
| | | 1376-1598 | OM993557 | KX671562 | R,M,C,**S**,3S | 3.39E−08 |
| | | 2754-272 | MK158208 | KX671562 | **R**,G,B,M,C,S,3S | 9.63E−12 |
| ON054966 | 1 | 1497-2655 | KX671562 | Unknown | B,M,C,**S** | 6.06E−16 |

**Table S2.** Recombination events detected in PeLCV-Pak

| PeLCV accession # | Number of recombination events | Recombination breakpoint* | Major parent | Minor parent | RDP methods | Highest P values detected by the underline method# |
|---|---|---|---|---|---|---|
| AM948961 | 1 | 1990-2574 | MN566098 | Unknown | R,G,M,C,S,**3S** | 1.85E−11 |
| DQ116884 | 2 | 978-2561 | KY937947 | LN678638 | R,**M**,C,S,3S | 5.86E−08 |
| | | 2577-2622 | KY937947 | Unknown | R,G,M,**C** | 1.31E−04 |
| HF568781 | 1 | 1169-2751 | OM993557 | LT600729 | R,G,B,M,S,**3S** | 2.43E−36 |
| KX671561 | 1 | 1169-2751 | OM993557 | LT600729 | R,G,B,M,S,**3S** | 2.43E−36 |
| KX671562 | 1 | 1169-2751 | OM993557 | LT600729 | R,G,B,M,S,**3S** | 2.43E−36 |
| KX671563 | 2 | 1171-1966 | MN885484 | Unknown | R,G,M,C,**3S** | 7.01E−18 |
| | | 1194-1830 | Unknown | KX671562 | R,B,M,**3S** | 2.13E−07 |
| KX710160 | 3 | 834-1075 | MN885483 | Unknown | **R**,G,B,M,C,S,3S | 8.79E−10 |
| | | 1921-2210 | LT795118 | MF278789 | G,M,**C**,S,3S | 2.06E−04 |
| | | 2559-648 | LN713272 | KY937947 | R,G,**M**,C,S,3S | 3.10E−09 |
| KX711622 | 1 | 1162-2750 | OM993557 | LT600729 | R,G,B,M,S,**3S** | 2.43E−36 |
| LN678638 | 1 | 1952-2400 | LT795118 | KY937947 | R,M,**S**,3S | 1.27E−26 |
| LN713272 | 1 | 1924-2572 | MN885484 | Unknown | R,B,M,**S** | 1.01E−08 |
| LN713273 | 1 | 1952-2400 | LT795118 | KY937947 | R,M,**S**,3S | 1.27E−26 |
| LT600729 | 1 | 2739-978 | KX671563 | Unknown | R,G,B,M,**S**,3S | 3.24E−14 |
| LT795117 | 1 | 2729-1142 | KX671563 | Unknown | R,G,B,M,**S**,3S | 3.24E−14 |
| LT795118 | 1 | 2729-1142 | KX671563 | Unknown | R,G,B,M,**S**,3S | 3.24E−14 |

| | | | | | | |
|---|---|---|---|---|---|---|
| MF135486 | 1 | 1135-2729 | OM993557 | LT600729 | R,G,B,M,S,**3S** | 2.43E−36 |
| MG764701 | 1 | 1924-2127 | KY671562 | KY937947 | R,B,M,**S**,3S | 1.19E−23 |
| MG764702 | 1 | 1924-2127 | KY671562 | KY937947 | R,B,M,**S**,3S | 1.19E−23 |
| MG764703 | 1 | 1924-2127 | KY671562 | KY937947 | R,B,M,**S**,3S | 1.19E−23 |
| MG764704 | 1 | 1298-2254 | KY671562 | KY937947 | R,B,M,**S**,3S | 1.19E−23 |
| MG764705 | 1 | 1924-2127 | KY671562 | KY937947 | R,B,M,**S**,3S | 1.19E−23 |
| MH550515 | 2 | 2515-272 | MK158208 | MN885482 | G,B,M,S,**3S** | 2.37E−09 |
| | | 2514-2777 | OM993556 | MK158208 | R,G,B,M,S,**3S** | 3.52E−23 |
| MH643736 | 2 | 1140-2480 | MF278789 | Unknown | R,M,S,**3S** | 1.44E−12 |
| | | 2745-352 | MF278789 | Unknown | R,M,S,**3S** | 4.51E−15 |
| MH973686 | 1 | 2-1037 | KX671563 | Unknown | R,G,B,M,S,**3S** | 2.98E−14 |
| MH973687 | 2 | 1661-1985 | LT600729 | Unknown | R,G,M,S,**3S** | 1.41E−09 |
| | | 2739-1139 | KX671563 | OM993556 | R,G,B,M,S,**3S** | 7.98E−29 |
| MH973688 | 2 | 4-1161 | KX671563 | OM993556 | R,G,B,M,S,**3S** | 7.98E−29 |
| | | 1746-1987 | LT600729 | Unknown | R,G,M,S,**3S** | 1.41E−09 |
| MK158207 | 2 | 1594-1946 | MN885482 | KY937947 | R,G,B,M,C,**S**,3S | 2.21E−08 |
| | | 25-267 | MF135486 | Unknown | M,C,**S**,3S | 7.33E−04 |
| MK158208 | 1 | 1496-2401 | MN885482 | KY937947 | R,G,B,M,C,**S**,3S | 2.21E−08 |
| MK158209 | 3 | 384-2147 | OM993555 | Unknown | R,G,B,M,C,S,**3S** | 21.57E−16 |
| | | 1484-1974 | OM993555 | Unknown | R,G,M,C,S,**3S** | 1.44E−11 |
| | | 2393-2696 | MH550115 | MH973686 | G,M,C,S,**3S** | 7.46E−27 |
| MN566097 | 2 | 381-511 | LN713272 | KY937947 | **R**,G,C,M,S,**3S** | 2.57E−09 |
| | | 1164-2753 | OM993557 | LT600729 | R,G,M,S,**3S** | 2.43E−36 |
| MN566098 | 3 | 381-511 | LN713272 | KY937947 | **R**,G,C,M,S,**3S** | 2.57E−09 |
| | | 1164-2753 | OM993557 | LT600729 | R,G,M,S,**3S** | 2.43E−36 |
| | | 1425-2525 | Unknown | MN885484 | B,M,S,**3S** | 4.46E−07 |
| MN885482 | 1 | 1952-2357 | LT795118 | KY937947 | R,B,M,**S**,3S | 2.04E−29 |
| MN885483 | 1 | 1914-2527 | MN885484 | Unknown | R,G,B,M,S,**3S** | 9.86E−10 |
| MN885484 | 2 | 37-381 | LT600729 | MF135486 | R,G,B,C,M,S,**3S** | 1.53E−25 |
| | | 2725-1101 | KX671563 | Unknown | R,G,B,C,M,**S**,3S | 3.24E−14 |
| MN885485 | 1 | 59-1101 | KX671563 | Unknown | R,G,B,C,M,S,**3S** | 3.24E−14 |
| MN910265 | 2 | 302-812 | KY937947 | Unknown | R,G,C,M,S,**3S** | 1.35E−12 |
| | | 2120-2443 | KY937947 | LN678638 | R,**C**,M,S,3S | 2.16E−07 |
| OM993555 | 3 | 1486-1972 | MN885482 | KY937947 | R,G,B,C,M,**S** | 2.21E−06 |
| | | 2349-2724 | MH550115 | MH973687 | G,C,M,S,**3S** | 7.46E−28 |
| | | 2349-253 | MK158208 | MF278789 | R,G,B,C,M,**S**,3S | 1.12E−16 |
| OM993556 | 3 | 615-1518 | Unknown | LN713272 | B,C,M,**S** | 2.70E−14 |
| | | 1535-2407 | MN885482 | KY937947 | R,G,B,C,M,**S**,3S | 2.21E−08 |
| | | 2731-271 | MK158208 | MN885482 | G,B,C,M,S,**3S** | 2.36E−09 |
| OM993557 | 2 | 2513-2765 | OM993556 | MK158208 | R,G,B,C,M,S,**3S** | 3.52E−23 |
| | | 2513-348 | MK158208 | MN885482 | G,B,C,M,S,**3S** | 2.36E−09 |

| | | | | | | |
|---|---|---|---|---|---|---|
| NC008299 | 2 | 1076-2576 | KY937947 | LN678638 | R,**M**,C,S,3S | 5.86E−08 |
| | | 2577-2642 | KY937947 | Unknown | R,G,M,**C** | 1.74E−08 |

**Table S3.** Recombination events detected in PeLCV-Ind

| PeLCV accession # | Number of recombination events | Recombination breakpoint* | Major parent | Minor parent | RDP methods | Highest P values detected by the underline method# |
|---|---|---|---|---|---|---|
| JN807764 | 3 | 93-573 | MH765695 | OK236813 | R,G,B,M,**C**,S,3S | 1.08E−08 |
| | | 1074-1654 | JQ012792 | Unknown | R,G,B,M,C,S,**3S** | 7.55E−13 |
| | | 1734-2518 | KX168427 | OK236813 | R,B,M,C,**S**,3S | 1.91E−15 |
| JQ12790 | 3 | 93-573 | MH765695 | OK236813 | R,G,B,M,S,**3S** | 2.26E−10 |
| | | 1076-1865 | KX168427 | OK236813 | R,G,B,M,C,S,**3S** | 9.01E−08 |
| | | 1962-2563 | KX168427 | OK236813 | R,B,M,C,**S**,3S | 2.48E−15 |
| MH765695 | 1 | 1076-2548 | JQ012790 | Unknown | R,B,M,C,**S**,3S | 9.92E−25 |
| OM144969 | 2 | 1261-2425 | OK236813 | Unknown | R,G,B,M,C,S,**3S** | 4.44E−14 |
| | | 2740-74 | OK236813 | KX168427 | R,G,B,**M**,C,3S | 1.96E−07 |
| KX168427 | 3 | 80-596 | MH765695 | OK236813 | R,G,B,M,C,S,**3S** | 2.26E−10 |
| | | 1130-1324 | MH765695 | OK236813 | G,M,C,**S**,3S | 3.87E−10 |
| | | 1562-1879 | MH765695 | OM144969 | R,M,C,**S**, | 5.97E−16 |
